# A decision theory paradigm for evaluating identifier mapping and filtering methods using data integration

**DOI:** 10.1186/1471-2105-14-223

**Published:** 2013-07-15

**Authors:** Roger S Day, Kevin K McDade

**Affiliations:** 1 Department of Biomedical Informatics, University of Pittsburgh School of Medicine, Pittsburgh, USA; 2 Department of Biostatistics, University of Pittsburgh Graduate School of Public Health, Pittsburgh, USA

## Abstract

**Background:**

In bioinformatics, we pre-process raw data into a format ready for answering medical and biological questions. A key step in processing is labeling the measured features with the identities of the molecules purportedly assayed: “**molecular identification**” (**MI**). Biological meaning comes from identifying these molecular measurements correctly with actual molecular species. But MI can be incorrect. **Identifier filtering** (**IDF**) selects features with more trusted MI, leaving a smaller, but more correct dataset. **Identifier mapping** (**IDM**) is needed when an analyst is combining two high-throughput (HT) measurement platforms on the same samples. IDM produces ID pairs, one ID from each platform, where the mapping declares that the two analytes are associated through a causal path, direct or indirect (example: pairing an ID for an mRNA species with an ID for a protein species that is its putative translation). Many competing solutions for IDF and IDM exist. Analysts need a rigorous method for evaluating and comparing all these choices.

**Results:**

We describe a paradigm for critically evaluating and comparing IDF and IDM methods, guided by data on biological samples. The requirements are: a large set of biological samples, measurements on those samples from at least two high-throughput platforms, a model family connecting features from the platforms, and an association measure. From these ingredients, one fits a mixture model coupled to a decision framework. We demonstrate this evaluation paradigm in three settings: comparing performance of several bioinformatics resources for IDM between transcripts and proteins, comparing several published microarray probeset IDF methods and their combinations, and selecting optimal quality thresholds for tandem mass spectrometry spectral events.

**Conclusions:**

The paradigm outlined here provides a data-grounded approach for evaluating the quality not just of IDM and IDF, but of any pre-processing step or pipeline. The results will help researchers to semantically integrate or filter data optimally, and help bioinformatics database curators to track changes in quality over time and even to troubleshoot causes of MI errors.

## Background

A key step in preparing raw high-throughput (HT) data into analyzable data suitable for answering medical and biological questions is labeling the measured features with the identities of the molecular species purportedly assayed: “molecular identification” (MI). There are many ways to get these MIs, thanks to a proliferation of annotation databases and algorithms. MI may come from (a) an online annotation resource (example: Ensembl/EnVision from ENFIN [1]), (b) an annotation data object in a bioinformatics-capable software environment (example: one of bioconductor’s “annotationDBI” objects [2]), or (c) an algorithm (examples: Sequest [3] in proteomics or Tophat [4] for short read alignments in RNA-Seq). If the MI process is done well, then the analysis of HT biological data has the best chance of yielding meaningful results.

Incorrect MIs appear to be frequent [5]. It is common for two well-respected MI methods or resources to diverge substantially [6]. Thus many MIs are likely to be incorrect. If so, then any analysis which depends on biological interpretation risks missing or mischaracterizing an important discovery , because biological interpretation depends on correctly identifying the assessed molecular species.

In choosing MI methods, HT data analysts have limited guidance. Which techniques they use for filtering and mapping are typically based on convenience, habit, and heuristics. Further, research articles seldom report which techniques they chose. When their choices are data-driven, the same data set under scientific study is also used to assess the choices, leading to potential biases.

To improve MI, we need to measure its quality. The approach promoted here provides a “measuring instrument” for the quality of an MI method, an instrument to be used for comparing MI methods and for tracking changes in MI quality over time.

One kind of MI is identifier filtering (IDF): deciding which features in a data set to include and which to exclude based on some believability criterion, whether computed or curated. Each HT platform produces a large set of features; each feature is labeled by an identifier (ID). Prior filtering of features is especially important in light of the multiple testing quandary, which is aggravated when many incorrect features compete with the genuine features for the attention of a statistical testing procedure. There are numerous criteria for filtering out less believable features, but the practical question is which single IDF criterion is best, or how to construct a combination better than any single one.

Another kind of MI is mapping between the identifiers from two platforms: ID mapping (IDM). More than ever, bioinformatics analysis involves integrating data from multiple platforms. Data integration across platforms is needed for a sufficiently full picture of biological systems. But the term “data integration” has multiple meanings. Integrating HT data from multiple platforms on the same samples can occur at three levels.

### a) Separate analyses

Many studies report combined analyses across assay platforms, but the data integration consists of reporting independent separate analyses of each platform, culminating in combining the results of each in simple ways, for example, a comparison of biomarker candidate lists [7] or interpretations of modulated features [8]. This kind of study does not require merging the data by sample. Good MI is nevertheless important in these studies, because good identifier filtering (IDF) will remove misidentified features from the data, decreasing false discoveries and increasing detection of true discoveries.

### b) Merged by sample

One can also merge data by sample to create a combined collection of measured molecular features. Sample-wise merging enlarges the feature set by obtaining features from both platforms. Some studies develop predictive or prognostic models utilizing these enlarged sets [9,10]. No IDM is required. Again, good IDF is important.

### c) Merged by sample and semantics

To generate deeper understanding and fulfill the promise of systems biology, the data need to be merged not just by sample, but by biological meaning as well: semantic merging. This requires identifier mapping, IDM, to connect the MIs annotating measurements on two different platforms.

The resources that do algorithm-based and annotation-based IDM are numerous ([11-14] and many others). But they disagree with each other. They may even disagree with themselves, comparing results based on web service queries to results based on file downloads, or comparing one year to another [6]. When undertaking a sophisticated systems analysis performed on a multiple-platform data set, incorrect MIs will at best complicate analysis, and at worst obscure the truth and sabotage the chance to build meaningful systems biology models.

The paradigm we present here give us a way to interrogate the quality of the IDM and IDF methods in the context of biological material. This approach uniquely fills a major gap in the effort to improve bioinformatics analysis. After presenting examples, its potential range of application is discussed.

## Methods

We present a general strategy to compare quality of data preparation strategies. The essential ingredients are:

• A substantial number of ***biological samples*** (roughly 100 or more).

• ***Two bioinformatic HT datasets*** measuring related classes of molecules, such as mRNAs and proteins, or microRNAs and mRNAs, or gene copy number and mRNAs, on the same set of samples.

• A ***set of candidate methods*** for ways to accomplish IDM or IDF. The result of each candidate *M* is a set ***S***(***M***) of ID pairs accepted by *M*. Candidates can be composites of other candidates: for example strategies combining one IDM and one IDF strategy, or Boolean combinations of multiple IDMs or IDFs.

• An IDM method to create the full pool of ID pairs. When the candidate methods themselves are IDM methods, the full pool is just the union of the sets of pairs from the candidate IDM methods.

• A simple ***target model***, relating the measurements, to be applied separately for each ID pair (***P***) of features in the full ID pair set. This will typically be a regression model, where the primary feature is to be predicted by the secondary features. The predictor feature list may be augmented by auxiliary features, such as sample categories. In the examples, the simple model is that, for each pairing of transcript and putative protein product, protein abundance is proportional to (or at least monotonically related to) transcript abundance when MI and IDM are correct, but unrelated when it is not.

• A ***model quality score***, notated as ***MQ***(***P***), for each ID pair *P*. In the applications described below, *MQ* is the correlation of the two features.

In the case of mapping mRNA expression and protein expression, we expect a correctly mapped and biologically coupled feature pair to manifest in a strong positive correlation. In other cases, a correctly mapped pair might manifest as a strong negative correlation, for example when the pair consists of a microRNA species and a transcript that it putatively downregulates.

We form the collection of all scores *MQ****(****P****)*** for the union of ID pairs from all the candidate methods *M*: ∪_*M*_{*MQ*(*P*) : *P* ∈ *S*(*M*)}. This list of *MQ* scores can be analyzed immediately via regression modeling, visualization, and two-sample tests, to see which of the methods *M* are most competent at *MI*.

### The mixture model for evaluation of resources

**Figure 1:**
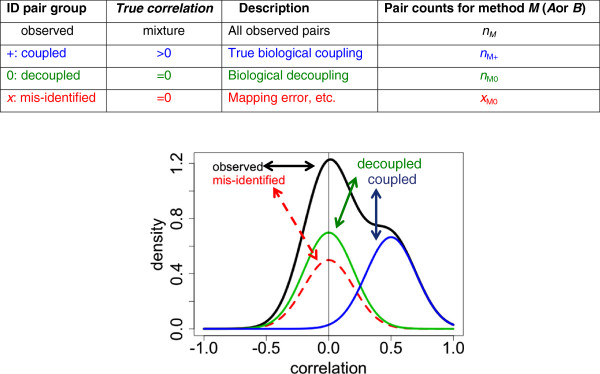
**Hypothetical mixture components for correlation.** Observed (black): marginal density of correlations. Mis-identified (red, dotted): density of correlations where either feature is mis-identified, or they are incorrectly mapped. Decoupled (green): density of correlations of pairs correctly mapped but biologically uncorrelated (“discordant”). Coupled (blue): density of correlations of pairs correctly mapped and biologically coupled.

However, the enterprise is more effective if we first convert the *MQ* scores into posterior probabilities. Figure 1 illustrates the key argument. The central concept is that pairs of ostensibly related features from two different platforms should fall into three categories:

“**+**”: both features are correctly identified and mapped between platforms, and truly biologically ***coupled*** (for example, correlated) as expected,

“**0**”: both features are correctly identified and mapped, but biologically ***decoupled*** in the sample group under study, due to causes unaccounted-for up to now, so that the correlation, regression coefficient, or other measure of association is near zero. This decoupling is sometimes called discordance.

“***x***”: one or both features are ***incorrectly*** identified, or incorrectly mapped to each other.

Comparing two methods *A* and *B* for IDF or IDM, the best of the two should be relatively enriched for the “+” and “0” categories of feature pairs. With enough samples and enough feature pairs, the identity of the best method should become clear.

The “0” group deserves further discussion. These pairs have correct MI, but additional biological factors decouple the two identified molecules. These extra factors may be as simple as unaccounted-for sources of variation in the target feature, lowering the signal to noise ratio. For example, in relating transcript to protein, true biological decoupling could occur, because of variation in rates of protein degradation, post-translational modification, and other factors affecting protein abundance as detected by mass spectrometry. Decoupling can stem from more exotic causes. Variation in microRNA might interfere with translation in a way that does not manifest itself in the raw abundance of the transcript. Feedback loops may buffer or magnify variation in transcript. There could be variation in transcript splicing, or in the time delay between transcription and translation. In measuring transcript expression cross-hybridization of other transcripts with the probesets targeting the genuine transcript could vary across subjects. Further discussion of decoupling and its causes is in Day 2011 [6].

On the level of individual targets, a single dataset cannot directly distinguish true decoupling, “0”, from incorrect MI, “*x*”. However, by aggregating tens of thousands of targets, typical for the HT platforms being merged, the ensemble of models and scores will overcome the signal-to-noise problem by volume of the number of identifier pairs, and help us find the MI performance differences we seek.

The following simple argument motivates the claim that the observed proportions of strong correlations reflect the relative quality of *A* and *B.* Our notation for the counts in the three groups is: are coupled and correctly mapped by *A,* are correctly mapped by *A* but not coupled, and are incorrectly mapped. If the true proportion in the “+” group is greater for *A* than *B,* then

(1) $$\frac{n_{A+}}{n_{A+}+n_{A0}+x_{A}}>\frac{n_{B+}}{n_{B+}+n_{B0}+x_{B}}.$$

As the number of samples increases, the measurement error in assessing whether a single pair is in the “+” group decreases. With sufficient numbers of ID pairs, determining which proportion in (1) is larger becomes more accurate. Suppose also that, given that a pair is correctly mapped, the coupling/decoupling status (true correlation) is independent of the method, so that

(2) $$n_{A+}/\left(n_{A+}+n_{A0}\right)=n_{B+}/\left(n_{B+}+n_{B0}\right)$$

is a constant *δ*_+_. Then the proportion of correct MI is greater for method *A*:

(3) $$\frac{n_{A+}+n_{A0}}{n_{A+}+n_{A0}+x_{A}}>\frac{n_{B+}+n_{B0}}{n_{B+}+n_{B0}+x_{B}}.$$

When (2) is true as well as (1), the ratios of the two sides are the same in (3). The same conclusion holds if, instead of (2),

(4) $$n_{A0}/\left(n_{A0}+x_{A}\right)=n_{B0}/\left(n_{B0}+x_{B}\right)$$

is a constant *δ*_0_, so that the proportion of correct mappings among those either decoupled or incorrect is the same for *A* and *B*. Note that conditions (2) and (4) are not equivalent. In summary, under mild assumptions, the resource with a larger observed proportion of good correlations is also the better mapping resource, and the ratio of the two sides in (1), which can be observed, is a consistent estimator for the ratio of the two sides of (3), the proportions of correct pairs.

### Mixture model for model quality score (*MQ*)

Our next step is to make the argument more rigorous and subject to quantitative analysis. Suppose that we have chosen an association measure *MQ*, for use as a measure of the likely correctness of an ID pair. (For convenience of exposition, we use “correlation” in this argument, but sometimes other measures may be preferable.) Suppose the observed correlation for pair k depends on the correctness of the mapping as follows:

(5) $$\begin{array}{c} \mathrm{MQ}\left(p\right)\sim N\left(\psi_{G\left(p\right)},\tau_{G\left(p\right)p}\right)\\ \;\text{where}\;\tau_{G\left(p\right)p}=\sigma_{p}^{2}+V_{G\left(p\right)} \end{array}$$

(6) $$\mathrm{Pr}\left(G\left(p\right)=g\right)=\pi_{g}\;\mathrm{for}\;g\;\in \left\{"+","0","x"\right\}$$

where *G*(*p*) indicates the group for pair *p* as defined above. We assume that ; that is, unless the pair is correct and coupled (“+”), its *MQ* has mean zero. The measurement error variances σ_p_^2^ are presumed known or estimated by bootstrap. The group variances *V*_+_, *V*_0_, *V*_*x*_ are unknown. From the observational point of view, the “0” and “*x*” groups are indistinguishable, so define *π*_−_=*π*_0_+*π*_*x*_ and *V*_−_=(*π*_0_*V*_0_+*π*_*x*_*V*_*x*_)/*π*_−_. We can now estimate the unknowns *ϕ*=(*ψ*_+_, *π*_+_, *V*_−_, *V*_+_) easily by an ECM algorithm [15] (Additional File 1), constraining *ψ*_−_=*ψ*_0_=*ψ*_*x*_= 0, to yield the maximum likelihood estimate $\hat{\phi }=\left({\hat{\psi }}_{+},{\hat{\pi }}_{+},{\hat{V}}_{-},{\hat{V}}_{+}\right)$.

Next, we can estimate the probability of each pair belonging to the “+” group, using the empirical Bayes “plug-in” technique, which replaces the parameters by their EM estimates. Defining $\pi_{+p}^{*}=\mathrm{Pr}\left(G\left(p\right)="+"|\mathrm{MQ}\left(p\right),\hat{\phi }\right)$ and $\pi_{-p}^{*}$ = 1 − $\pi_{+p}^{*}$, the posterior odds are

(7) $$\begin{array}{l} \frac{\pi_{+p}^{*}}{\pi_{-p}^{*}}=\frac{\mathrm{Pr}\left(G\left(p\right)="+"|\mathrm{MQ}\left(p\right),{\hat{\psi }}_{+},{\hat{V}}_{+},{\hat{\pi }}_{+}\right)}{\mathrm{Pr}\left(G\left(p\right)="-"|\mathrm{MQ}\left(p\right),{\hat{\psi }}_{-},{\hat{V}}_{-},{\hat{\pi }}_{-}\right)}\\ \;=\frac{{\hat{\pi }}_{+}}{{\hat{\pi }}_{-}}\frac{\exp\left(-{\left(\mathrm{MQ}\left(p\right)-{\hat{\psi }}_{+}\right)}^{2}/2\left({\hat{V}}_{+}+\sigma_{p}^{2}\right)\right)/\sqrt{{\hat{V}}_{+}+\sigma_{p}^{2}}}{\exp\left(-{\left(\mathrm{MQ}\left(p\right)-{\hat{\psi }}_{-}\right)}^{2}/2\left({\hat{V}}_{-}+\sigma_{p}^{2}\right)\right)/\sqrt{{\hat{V}}_{-}+\sigma_{p}^{2}}}. \end{array}$$

In addition, we gain an estimate of the accuracy of this posterior probability, in the form of a posterior variance, approximated using the delta method to convert from the bootstrap estimate of the sampling variance of the correlation to an estimate of the variance of the posterior probability:

(8) $$\begin{array}{l} v_{+p}^{*}=v\hat{\text{a}}r\left(\pi_{+p}\right)\\ \;≐\sigma_{p}^{2}{\left(\pi_{+p}^{*}\pi_{-p}^{*}\right)}^{2}{\left(\frac{\mathrm{MQ}\left(p\right)-{\hat{\psi }}_{-}}{{\hat{V}}_{-}+\sigma_{p}^{2}}-\frac{\mathrm{MQ}\left(p\right)-{\hat{\psi }}_{+}}{{\hat{V}}_{+}+\sigma_{p}^{2}}\right)}^{2}. \end{array}$$

Thus for each ID pair *p*, the mixture model provides a data-determined estimate of the probability that it is a correct annotation, and a measure of uncertainty for that probability. These ingredients provide what we need to decide which of two methods for ID mapping to use. (The case of filtering is a little more complicated, because then any two pairs that share the same ID subject to filtering also share their fate).

We can contrast this use of mixture models to Schaefer et al’s study [16] using a mixture model to examine concordance of ChIP-chip and ChIP-seq data, using data from just two samples. Aside from the difference in the number of samples studied, Schaefer’s purpose also was different: to characterize the concordance and discordance of the platforms. This contrasts with the purpose of our paradigm, to evaluate ID mapping and filtering methods.

### Expected utility to inform selection of best practices

In search of best practices, we want to choose between the ID pairs provided by method *M*=*A* or method *M*=*B.* A simple estimate of the proportion *P*_+*M*_ of correct ID pairs is just the mean of the  over the ID pairs from method *M*. An optimally weighted mean

(9) $${\hat{P}}_{+M}=\Sigma_{p\in S\left(M\right)}\pi_{+p}^{*}{\left(v_{+p}^{*}\right)}^{-1}/\Sigma_{p\in S\left(M\right)}{\left(v_{+p}^{*}\right)}^{-1}$$

is a more accurate estimate; it takes into account the variation in standard errors. This is important, since some correlations are highly unstable, for example due to a low variance in either protein or transcript expression. These will manifest as having middle-range values for $\pi_{+p}^{*}$, far from 0 or 1. Now we choose a value for *L*_*FP*_=the loss associated with a “false positive”, and *U*_*TP*_=the utility of including a “true positive”. With this estimate, one can choose whether to reject the entire subset of probesets produced by method *M* using a standard Bayesian expected loss calculation:

(10) $$\begin{array}{l} EU^{\left(1\right)}=E\left(\mathrm{utility}\;\mathrm{per}\;\mathrm{pair}|\mathrm{method}\;M\right)\\ \;=\Sigma_{p\in S\left(M\right)}\left(\pi_{+p}^{*}U_{\mathrm{TP}}\;-\;\pi_{-p}^{*}\;L_{\mathrm{FP}}\right){\left(v_{+p}^{*}\right)}^{-1}/\Sigma_{p\in S\left(M\right)}{\left(v_{+p}^{*}\right)}^{-1}\\ \;=U_{\mathrm{TP}}P_{+M}-L_{\mathrm{FP}}P_{-M} \end{array}$$

(11) $$\text{EU}\;\mathrm{total}=\mathrm{Total}\;\mathrm{expected}\;\mathrm{utility}|\mathrm{method}\;M=n_{M}EU_{M}^{\left(1\right)}$$

The break-even point is *P*_+*M*_=*L*_*FP*_/(*U*_*TP*_+*L*_*FP*_). We can choose method *M*=*A* or *B* based on which makes either the average or the total expected utility the largest, depending on the purpose. The posterior probabilities from (7) utilize as a prior the overall component probability as estimated by the generalized EM algorithm; this can be modified to a true Bayesian prior if desired. We also can take into account that some proportion of the correctly included or mapped pairs are in group “0”. One data set standing alone cannot distinguish “0” from “−”. However, if the analyst is willing to speculate on the ratio introduced in (2), then letting *δ*_+_=*n*_*M* +_/(*n*_*M*0_+*n*_*M* +_) we can modify (10) by adding an extra term accounting for the appearance of some correct pairs in the “−” group:

(12) $$\begin{array}{l} EU^{\left(1\right)}=\left(U_{\mathrm{TP}}+L_{\mathrm{FP}}\right)P_{+M}\;-\;L_{\mathrm{FP}}+(U_{\mathrm{TP}}-L_{\mathrm{FP}})P_{+M}(\delta_{+}^{-1}-1)\\ \;=P_{+M}\left(U_{\mathrm{TP}}\delta_{+}^{\;-1}+L_{\mathrm{FP}}\left(2-\delta_{+}^{-1}\right)\right)\;-\;L_{\mathrm{FP}} \end{array}$$

which is just an increasing linear function of *P*_+*M*_. If our criterion is *EU total* (11), a more stringent method may improve *P*_+*M*_ but decrease the number of pairs returned, *n*_*M*_, enough to reverse the optimal choice. This gives the analyst a basis to choose a more generous method, if it benefits the analytical purpose.

## Results

We illustrate the paradigm with two distinct evaluations of MI (one for IDF and one for IDM) and one evaluation of threshold choice, all using the same pair of HT datasets for samples in an endometrial cancer setting. A full description of the data is published [6]. Briefly, in a study of analyses of 91 endometrial cancer and 7 noncancer endometrial samples, liquid chromatography-tandem mass spectrometry generated 11,879 distinct UniProt accessions, and the Affymetrix U133 Plus 2.0 microarray provided expression data on the same samples. In a previous report, we utilized these data to compare the quality of three annotation resources, DAVID [17-20], EnVision [1,21], and NetAffx [22,23], for mapping identifiers between Affymetrix probeset IDs and UniProt accession IDs, selected by virtue of providing direct mappings of probesets.

The analysis begins by utilizing only the ID maps retrieved from each resource. We comprehensively compared the retrieved sets of ID maps. For example, less than half (45.3%) of the UniProt protein accessions pulled identical lists from NetAffx and EnVision. Similar results were obtained comparing each of NetAffx and EnVision with DAVID.

**Figure 2:**
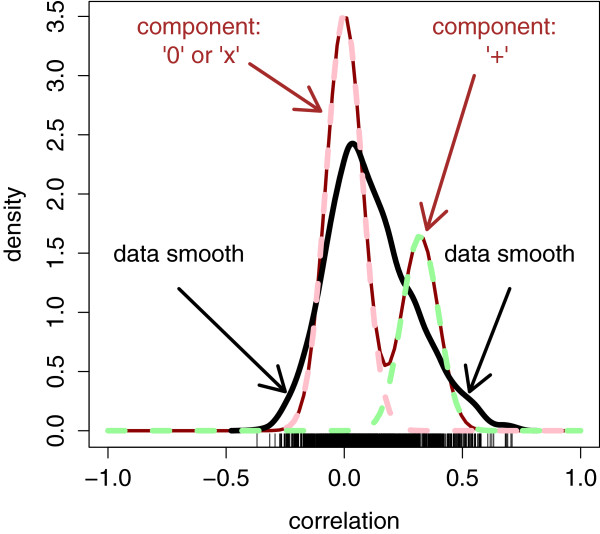
**Distribution and mixture fit of observed correlations.** Black curve: a crude empirical density smooth for the observed correlations, which correspond to the “rug” whiskers along the bottom. Brown bimodal curve: the mixture fit to the underlying “true” correlation distribution, marginalized over the mixture component. Pink and green dotted curves: the mixture components, multiplied by their probabilities. Pink: decoupled (“0”) or mismatched (“*x*”). Green: coupled and correctly mapped (“*+*”).

For each IDM pair (UniProt, probeset), the data were merged by sample and the correlation between the two platforms computed. We proceeded under the presumption that better identifier matching should generate a higher rate of match pairs with strong correlations between the transcript signals and corresponding protein spectral counts. We decomposed the correlations, as described above, into a mixture with a zero-centered component and a positive component (Figure 2). The left-most component can be interpreted as our “–” group: a mixture of our “0” and “*x*” groups, which cannot be distinguished empirically at this point; the second as our “+” group. The mixture distribution model sheds light on the degree to which positive correlations exceed negative ones, allowing estimation of the distribution of correlations among correctly mapped protein-probeset pairs, without needing to know at this point which specific pairs are correctly mapped and which are not. It also provides a measure more useful than the correlation itself: the posterior probability of belonging to the right-hand mixture component. This component (green dotted line) corresponds to the “+” group: probeset-protein ID pairs which are both correctly identified and mapped, and biologically correlated**.**

**Figure 3:**
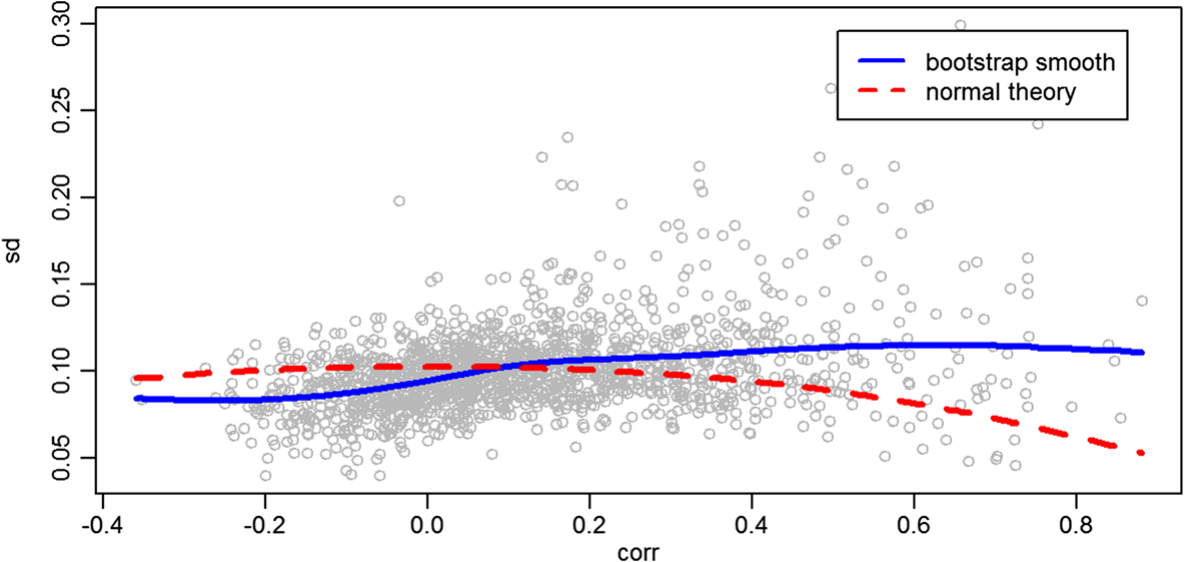
**Bootstrap estimates of standard deviation of the correlations.** Each point shows the Pearson correlation between features of an ID pair, versus the square root of its bootstrap variance estimate (R=200 replications). The blue solid line is a loess smooth of these points. The red dotted line is from the normal theory expression for the variance (1?−?*ρ*^2^)/(*n*?−?3) of a Pearson correlation coefficient estimate $\hat{p}$. The smooth fit for the relationship between the correlation and the bootstrap standard deviation follows the normal theory curve well except at large values, but the individual bootstrap estimates vary from the curve substantially.

**Figure 4:**
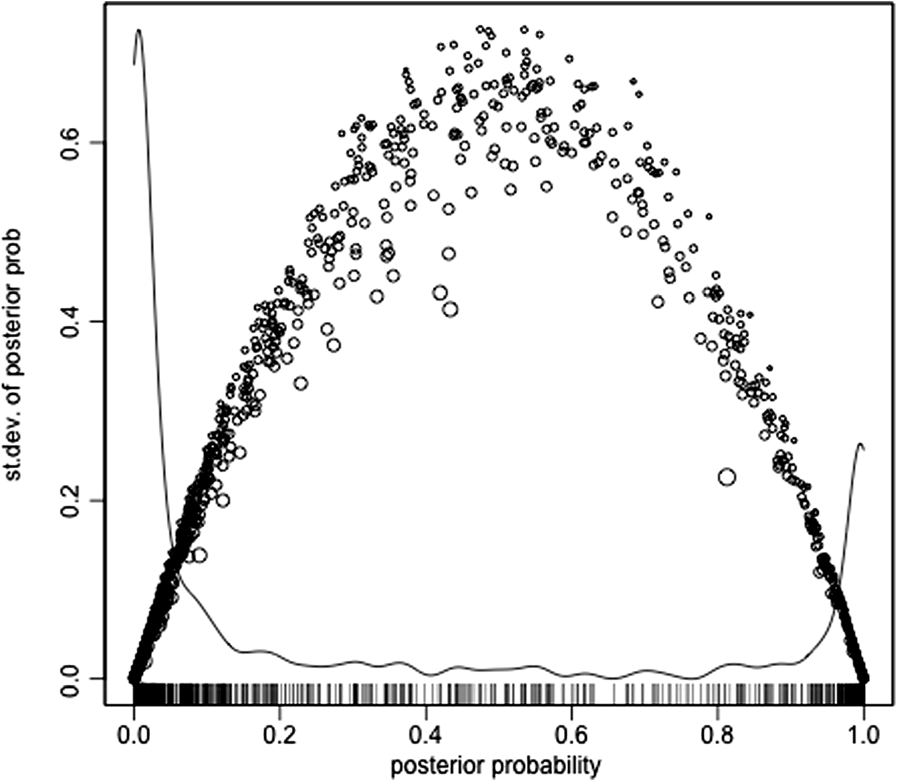
**Relationship between posterior probability and posterior standard deviation.** The sizes of the circles are proportional to the measurement standard deviation. The curve is a density estimate for the posterior probability.

Note that the empirical density smooth (black curve) is raw; it summarizes the observed correlation distribution. In contrast, the bimodal mixture fit (blue curve) obtained from the EM algorithm estimates the true underlying correlation distribution. It properly takes into account the measurement variances, estimated via individual bootstraps, to deconvolve (“unsmooth”) the observed distribution (black curve). It is not expected to approximate the density smooth unless the correlation measurement error is zero. Utilizing the bootstrap for estimating the measurement error variances *σ*_*p*_^2^ is important. In Figure 3 we see the bootstrap estimates plotted against correlation. Overall, the relationship echoes the normal-theory variance formula, but there is tremendous variability around that relationship, probably due to the nature of spectral count data. In Figure 4, we see the relationship between posterior probability and its posterior standard deviation. Ordinarily one expects large measurement standard deviations (obtained from the bootstrap) to associate with large posterior standard deviations. However, a large measurement standard deviation implies a paucity of information about the classification, a posterior probability far from zero or one, and an insensitivity of the posterior probability as the correlation value changes; this view of behavior with large *σ*_*p*_^2^ is confirmed by inspection of Equation (8) and Figure 4.

Next we examine the mixture from the decision theory perspective, in order to illuminate selection of best practices. In Table 1, the consequences of choosing an ID method are represented for a subset of ID pairs corresponding to proteins with a minimum average spectral count across the samples. The values guiding the decision analysis, chosen for illustration, are:

• *Utp*=2=utility of including into further analysis a “true” correctly mapped pair.

• *Lfp*=1=the loss, or negative utility, of including an incorrectly mapped pair.

• *δ*_+_=1=proportion of correctly mapped pairs which are coupled.

**Table 1:** Comparison of ID mapping resources

**ID pair group**	**nPairs**	**Pr(+)**	**Pr(−)**	**Utrue**	**Lfalse**	**EU mean**	**EU total**
EnVision	887	0.431	0.569	0.862	0.569	0.293	260
NetAffx	1147	0.380	0.620	0.761	0.620	0.141	162
DAVID	1401	0.335	0.665	0.671	0.665	0.006	8
Use All	1522	0.323	0.677	0.647	0.677	−0.030	−45

**Use All**=the union. ***Columns****: nPairs*=the number of UniProt-ProbeSet pairs reported by the indicated service; *Utrue*=the average utility from “+” and “0” pairs; *Lfalse*=the average loss from “−” pairs; *EU mean* is the average net utility per pair; *EU total*=*nPairs*?×?*EU mean*.

(Choosing *δ*_+_=1 means assuming as a working hypothesis that the “0” group of decoupled pairs is empty).

The all-or-none decision is appropriate when the purpose of the evaluation is to select a best practice for ID mapping, to be applied when integrating other data sets with the same feature identifiers. Since EnVision reports out fewer pairs, its relative standing declines if the criterion is the *EU total* instead of *EU mean*. Utilizing weighting reflecting relative measurement precision has a notable effect. In this case, without weighting DAVID slightly outperforms NetAffx (data not shown; consistent with the regression model in [6]), but utilizing the weighting, as in Table 1, reverses the ranking, though the difference is still small. The subjective elements in the decision include: *Utp, Lfp*, *δ*_+_ and whether to rely on *EU mean* or *EU total*. The relative ranking of the choices is usually insensitive to these elements.

To examine all reasonable ID mapping strategies, we need to include Boolean combinations (Table 2). Utilizing “*EU total*” as the criterion, the optimal strategy across individual and Boolean strategies is to keep ID pairs that Envision reports. Using “*EU mean*” (*EU*^(1)^), Table 2 would favor intersecting EnVision and DAVID pairs, but one must balance the improvement against the extra effort of retrieving an ID map from a second data source. In the rest of the paper, where we turn the focus away from ID mapping strategies, we take this conclusion into consideration, and utilize only the microarray/mass spectrometry ID pairs that EnVision provides to integrate the data.

**Table 2:** Boolean-defined ID mapping strategies

**ID pair group**	**nPairs**	**Pr(+)**	**Pr(−)**	**Utrue**	**Lfalse**	**EU mean**	**EU total**
N, E and D	853	0.434	0.566	0.867	0.566	0.301	257
N and E	1114	0.389	0.611	0.778	0.611	0.167	186
E and D	862	0.431	0.569	0.862	0.569	0.293	253
N and D	865	0.435	0.565	0.871	0.565	0.306	265
N or E	1434	0.330	0.670	0.660	0.670	−0.010	−14
E or D	1172	0.382	0.618	0.764	0.618	0.145	170
N or D	1423	0.335	0.665	0.671	0.665	0.006	9
N, E or D	1447	0.331	0.669	0.662	0.669	−0.007	−10

*Columns:* as in previous table. *Rows:* Boolean combinations of NetAffx-affirmed (N), EnVision-affirmed (E) and DAVID-affirmed (D) ID pairs.

### Assessing probeset filtering

Another application of the paradigm uses the same integrated proteotranscriptomic data, restricted to the EnVision-selected pairing, to evaluate the performance of probeset filtering mechanisms. (A comprehensive analysis of nine mechanisms is in final preparation. Next, we describe four of those methods for filtering out probesets, in chronological order of their introduction into use, and present their quality assessments alone and in combination.

“Affytag” remove probesets for which the Affymetrix ID contains a qualifier; that is, the ID ends in “_[agirxsf]_at”, reflecting original doubts concerning the correct and unique hybridization of the probes in each probeset, as documented by Affymetrix when the array was designed. Although the identifier tags were initially used as the ‘de facto’ quality measure, these tags were found to be unreliable [24]. We use the Affymetrix tags to evaluate historical probeset selection choices against more recent models, since an overwhelming number of studies have utilized these Affymetrix identifiers. We include it to provide a validation that the quality assessment paradigm can detect the expected deficiency of performance in a superseded method.

“Masker” [25] removes probesets omitted from the NCI “masked” chip description file. “PdbA30” removes probesets if more than 30% of probes are “bad” according to PLANdbAffy [26]. (The 30% figure is the result of an optimization step, not shown.) “Jetset” [27] calculates the product of a specificity score, a coverage score, and a robustness score, and removes probesets for which a higher-scoring probeset for the same Gene Symbol exists. The probeset acceptance rates among the 54613 probesets of the U133plus2.0 array are seen in Table 3:

**Table 3:** Probesets and ID pairs affirmed by selected ID filtering strategies

**Probeset**	**Probesets accepted**	**ID pairs accepted**	**Estimate**
**accepted by…**	**(of 54613)**	**(of 887)**^**1**^	**(P value**^**2**^**)**
Affytag	68.4%	39.2%	−0.04 ( NS)
Masker	92.5%	95.9%	0.24 (3.5e-07)
PdbA30	66.4%	74.2%	0.21 (1.1e-07)
Jetset	35.2%	48.9%	0.16 (2.6e-10)

^1^Pairs initially filtered as described above.

^2^Regression estimates are from a linear model predicting “+” group status with main effects only.

The most recent method, Jetset, has odds ratios (OR) in excess of 2.5 with both Masker (2.90) and PdbA30 (3.54), but much less with Affytag (1.24). Masker is roughly independent of PdbA30 and Affytag (OR = 0.92, 1.08). Finally, PdbA30 is inversely related to Affytag (OR=0.59). These results suggest that the filtering are not just repackaging the same insights, so strategies combining multiple methods deserve consideration.

**Figure 5:**
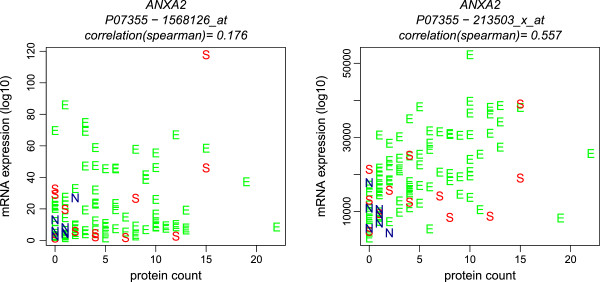
**Scatterplots of spectral counts versus microarray probeset signals.** Two probesets selected out of five mapped to the annexin 2 UniProt accession P07355. Symbols: N=non-cancer, S=serous carcinoma, E=endometrioid carcinoma. Figures are adapted from Day et al. [6].

Next, we introduce the integrated data to evaluate the methods and their Boolean combinations. Note that Jetset, PLANdbAffy, and other methods only use other bioinformatics resources to draw their recommendations. In contrast, our paradigm utilizes biological measurements on samples and requires the integration of biological data across platforms, in this case with a proteomic dataset. Therefore, it provides an independent validation using biological material. For illustration, Figure 5 shows scatterplots for spectral counts identified as annexin 2 (P07355) versus two probesets mapped to P07355. The leftmost shows a correlation of 0.176, low enough to question the mapping, while the rightmost correlation is 0.557, high enough to yield some confidence in the mapping, despite the fact that “Affytag” ID filtering would accept the left probeset and not the right. A single ID pair proves nothing; the aggregate across many tags is needed to learn anything about the quality of an ID filtering method.

Before we consider the decision-theoretic aspect, a look at a linear regression model to predict posterior probability of the “+” correlation group is revealing (Table 3). We assess the ID mapping and ID filtering choices together using the R function glm( ), with regression weights equaling the reciprocal of the variance estimates, 1/ v*_+*p*_, derived above via bootstrap and delta method. In this model, the terms for the NetAffx and DAVID ID mapping retrieval were nonsignificant, as was the term for the Affytag filtering method. The estimates were 0.15 for EnVision_Q (P=1.1e-06), 0.16 for Jetset (P=2.6e-10), 0.21 for PdbA30 (P=1.1e-17) and 0.24 for Masker (P=3.5e-07). This reinforces our decision to utilize the EnVision-mapped pairs only for evaluating filtering methods. Setting aside Affytag, then, we can look at second-order effects. Among the ID pairs approved by EnVision, the model was carried by the interaction term between PdbA30 and Jetset: estimate=0.247, P?<?0.0006) despite the large OR noted above. Even in the complementary dataset, containing only ID pairs disapproved by EnVision, the estimates are very similar, including the interaction term (0.284; P?<?0.0006).

This result reinforces the suggestion that the best practice is to utilize EnVision for ID mapping, and for ID filtering to utilize probesets which both Jetset and PdbA accept. It confirms the common view that the Affy tag, which dates to the origin of the array and represents knowledge from that time, is of little use for this purpose. Masker eliminates few probesets, but does provide significant extra value if Jetset is not used (data not shown).

Turning to the decision paradigm, we use the same subjective parameters as before, and generate the results in Table 4.

**Table 4:** Comparison of individual and Boolean combination filtering criteria for accepting probesets into an analysis dataset

**Accepted by…**	**nPairs**	**Pr(+)**	**Utrue**	**Lfalse**	**EU mean**	**EU total**
Affytag	348	0.493	0.986	0.507	0.479	167
PdbA30	658	0.518	1.04	0.482	0.553	364
Jetset	434	0.539	1.08	0.461	0.617	268
PdbA30 and Jetset	357	0.63	1.26	0.37	0.891	318
PdbA30 or Jetset	735	0.478	0.955	0.522	0.433	318
(No filtering)	887	0.431	0.862	0.569	0.293	260

These tables illustrate results with the same data and the same decision parameter settings as in the ID mapping example above. The results strongly suggest that the best strategy is to apply the PdbA filter alone if the criterion is *EU total*, but if the criterion is *EU mean* then by sacrificing quantity of pairs one can increase the average quality substantially by applying both the PdbA and the Jetset filters.

It is an important point that, while this conclusion depended on data integration, it is useful for any analyst dealing with data from the U133plus2.0 microarray, regardless of whether data integration with a proteomic or other platform is contemplated.

### Assessing threshold selection

We demonstrate the use of our paradigm for selecting thresholds in the context of filtering individual spectral events in tandem mass spectrometry. The purpose of presenting a threshold-related example is to demonstrate handling of a complication not seen in our previous examples: as the threshold changes, the actual data change, therefore the correlations and the bootstrap estimates of variance need recalculation. A common quality measure for spectral events is the cross-correlation, XCorr; another is the relative improvement of the best identification over the second-best, DeltaCn. For both measures, larger values should generally correspond to events with more reliable protein identifications. A reasonable question is whether the initial thresholds were stringent enough, or alternatively permitted too many erroneously identified spectral events.

In this example, we chose eight proportions (powers of ½ from 1 to 8) to use as potential thresholds. For each proportion, we then calculated the quantiles for XCorr and DeltaCn, then kept only the spectral events where both values exceeded the corresponding quantile. Next, we recalculated the correlations and variances, but kept the mixture model from the original data, using it to transform from correlation to posterior probabilityas we did in the previous examples.

**Figure 6:**
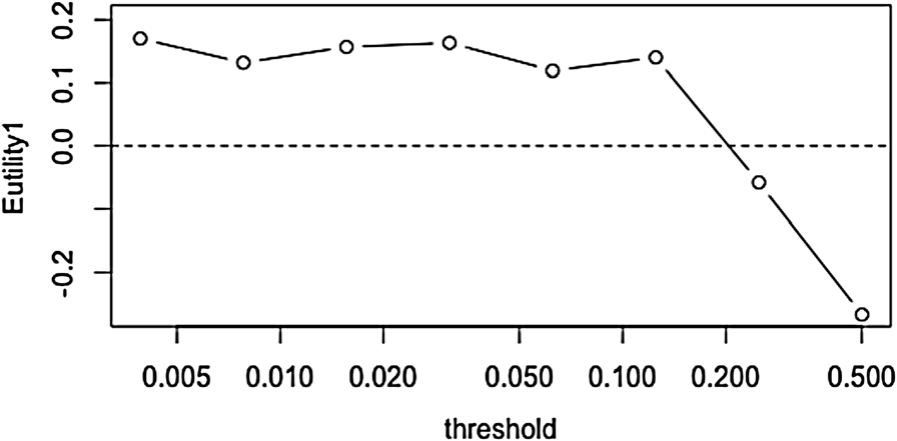
**Effect of spectral count filtering by threshold on average expected utility.** Horizontal axis shows the proportion to be excluded for the two spectral count criteria. Vertical axis show the mean expected utility (averaging across pairs).

Figure 6 and Table 5 show the results. As the threshold proportion changes, the thresholds for XCorr and DeltaCn change. Removing the lowest-quality spectral events did not improve the mean expected utility. (As the proportion of events removed exceeded 15%, the expected utility decreased, because as the sample counts decreased too much data was lost; some proteins lost all their events and exited the data set.) From these results, we conclude that the original thresholds for XCorr and DeltaCn were sufficiently stringent.

**Table 5:** Expected utility as quality threshold becomes more stringent

**Threshold**	**nEvents**	**nPairs**	**EU mean**	**EU total**
0	230948	887	0.20	181
0.00390625	229183	887	0.17	151
0.0078125	227459	887	0.13	117
0.015625	223922	887	0.16	139
0.03125	217116	887	0.16	145
0.0625	204335	887	0.12	105
0.125	181652	887	0.14	124
0.25	143940	887	−0.06	−51
0.5	84702	805	−0.27	−215

## Discussion

In this article, our examples focus on studies of best practices that can be obtained from integrating proteomic and transcriptomic data. We have demonstrated assessing and comparing methods for choosing an identifier mapping database, choosing an identifier filtering method, and setting an identifier filtering threshold. Note that, while our paradigm requires integrating data across platforms, identifier filtering applies to just one of the platforms, so the insights derived about identifier filtering methods can help even the data analyst working on that single platform. Furthermore, since every ID filtering evaluation depends on an ID mapping, when a better ID mapping method is found, it will lead to better ID filtering comparisons.

In comparing ID mapping methods, the methods do not need to be one-step mappings like the three we selected for the proteogenomic ID mapping study. In fact, the set of ID pairs do not need to consist of the same ID types, as long as they map features from the same pair of platforms. For example, a promising alternative is AbsIdConvert [28], which converts between ID domains by intermediate passage through mapping to a tree of genomic intervals, providing two-step indirect mappings and flexible, dynamic redefinition of probe-based features. The non-rigid mapping to genomic coordinates obviates many problems and potentiates some great applications. Applying AbsIdConvert to ID mapping between platforms requires an extra step that is not uniquely defined. Given mappings of two identifiers to genomic ranges, one must decide whether those ranges are “the same” for purposes of mapping between the the identifiers. Different rules for this decision generate different ID mapping methods, which are subject to our paradigm for comparative evaluation without special considerations.

With the decision framework, our evaluation paradigm can compare ID mappings even when the pairs are totally distinct. For Affymetrix arrays, many groups demonstrate methods to replace the probe sets by constructing new features, encoded in a chip definition file (CDF), with concomitant evidence of superiority [29-32]. In that case, each ID pair will belong to only one resource or method. The evaluation paradigm proceeds without change, with the mixture model utilizing all pairs just as above. The only substantive impact is that in fitting linear models predicting the posterior probability, like the one above that demonstrated a positive interaction between PdbA30 and Jetset, one can no longer estimate and test interaction terms.

It remains to consider to what extent the +/0/*x* classification could reflect reality. An incorrect mapping is an incorrect mapping, so the identity of the “*x*” group of ID pairs is conceptually clear-cut. However, the distinction between the coupled “+” and decoupled “0” groups can shift with context. First, an ID pair that is uncoupled in one data set might be coupled in another, reflecting differences in the biology in the two settings. Second, the dichotomous distinction is artificial. One might suppose instead that there is never total decoupling, just a variable amount of masking of the association, due to variation in the levels of known and unknown factors. However, the assumption in (2) seems plausible enough, since to our knowledge no mapping resources take into account results from data on sample.

### The timing of mapping resource retrieval

The proteogenomic integration supporting the examples presented here are from identifier mapping retrievals in 2011. In practice, an analyst will expect more recent retrievals to have higher quality. Subsequent releases of each resource do change; for example, from 2012 to 2013, NetAffx has changed the Uniprot match list for over 10% of probe sets in the HU133plus2.0 array. The staying power of best practices recommendations may exceed expectations; we have found no hint of a time trend in quality as measured by our correlation/posterior probability paradigm.

Nevertheless, analysts requirea convenient way to update best practices evaluations, especially for the large segment without R expertise. Our future plans call for a repository of integratable data set pairs from different platforms, and a web interface to make these method comparisons as easy and accessible and replicable as possible.

#### Wider applications

As long as there is data from two or more platforms on many samples, the paradigm we present can be applied to compare competing methods for virtually any data preparation step for either platform, filtering being just one example. We could use the same integrated protein expression/gene expression experiment described above to compare spectrum-to-protein [33,34] identification algorithms in “shotgun” mass spectrometry, and with nontraditional methods. For example, the Proteogenomic Mapping Tool [35] was proposed to improve genomic annotation, but can be repurposed to a new IDM method that might prove superior to the combination of spectral protein identification algorithm and ID mapping resource used in our examples here.

The paradigm also has a broad field of other applications, which belong to “ID mapping and filtering” only in an exended sense. Each of these presents special challenges, but no serious obstacles. One example arises in the setting of comparing segmentation algorithms for gene copy number data. Another example is comparing gene expression measures from expression arrays and RNA-seq, to clarify the strengths weaknesses and perhaps complementary nature [36] of the data from each. Among the many processing decisions in these studies, one is how to link the array probes or probe sets to genomic regions to which RNA-seq reads are aligned. Another application is comparing mRNA target algorithms for microRNA species, using correlation with expression. This can be considered a more complex form of ID mapping. However, it is more challenging, since the art of modeling the relationship between the entire complement of microRNA species and the expression of a target gene expression is in its infancy, and highly complex. Due to the multiple targets of microRNAs, a proposed microRNA regulator could have indirect effects with positive or negative correlations to the putative target transcript. However, the prior argument goes through if indirect effects cause positive or negative correlations equally, or less stringently if any asymmetry in the correlations is independent of whether method *A* or *B* produces the pair.

#### Bioconductor packages for evaluation of ID mapping and filtering

The examples described here utilize the IdMappingAnalysis [37] and IdMappingRetrieval [38] packages. IdMappingAnalysis provides a set of functions to compare the performance of selected ID mapping and filtering methods, using correlation across the samples between two measurements of features defined by the primary and secondary IDs. The new functionality presented in this article is now incorporated into version 1.5.1 of IdMappingAnalysis [39], available through bioconductor 2.13 development branch. These include the ECM algorithm used for mixture model fitting, the delta method for the variances of the posterior probabilities, and the calculation of the expected utilities.

## Conclusions

The paradigm which we present here depends on integrating two data sets on the same samples, a set of samples large enough to estimate correlations accurately. It does not depend on any special nature of the data preparation steps being assessed, so its range is not limited to just ID mapping and ID filtering. It behooves one to repeat those assessments on other data set pairs, to study the generalizability of the conclusions. The Cancer Genome Atlas (TCGA) [40] is well-suited to advance both the need to check generalizability and the opportunity to optimize a variety of data preparation steps. TCGA provides data on many thousands of human tumor samples and over a dozen bioinformatics HT platforms. Therefore it provides a wealth of opportunity for developing a systematic catalog of such assessments of data preparation methods, and makes the assessments easily and routinely available to analysts. As one example, in TCGA there are multiple pairs of RNA-seq and expression microarray data sets. Alignment algorithms provide a way to link these data sets for correlation analyses of the kind described here. As another example, TCGA has reverse-phase proteomic experiments on 538 ovarian cancer samples with corresponding Affymetrix array results. In a forthcoming report, we describe the use of these data in devising and validating best practices for array ID filtering.

### Availability of supporting data

The gene expression data were obtained from Affymetrix U133 Plus 2.0 microarrays. The raw data .cel and .chp files are available through GEO in a MIAME compliant format (http://www.ncbi.nlm.nih.gov/geo/query/acc.cgi?acc=GSE17025) and were released December 13, 2011. The proteomic data were summed readings from two tandem mass spectrometers. Details of the sample preparation, instrumentation, and spectral identifications are in Maxwell et al. [41]. Details of the identifier mapping resources utilized are in Day et al. [6].

## Abbreviations

MI: Molecular identification; HT: High throughput; ID: Identifier; IDF: Identifier filtering; IDM: Identifier mapping.

## Competing interests

There are no competing interests to report.

## Authors’ contributions

RSD developed the methodology and software code. KKM developed the applications to ID filtering. All authors read and approved the final manuscript.

## Supplementary Material

Additional file 1 ECM algorithm for two clusters with constraints. Click here for file
