# Supplementary material for: A decision theory paradigm for evaluating identifier mapping and filtering methods using data integration
Source: BMC Bioinformatics. 2013 Jul 15;14:223. doi: 10.1186/1471-2105-14-223 (PMC3734162; doi:10.1186/1471-2105-14-223)
Supplement: Additional file 1 — ECM algorithm for two clusters with constraints. [file 1471-2105-14-223-S1.docx]

**ECM algorithm for two clusters, with constraints on the cluster means and variances, and known data variances.**

Assume that

$$Y_{k}\sim N(\psi_{G(k)},\tau_{G(k)k})\text{ where }\tau_{G(k)k}=\sigma^{2}_{k}+V_{G(k)}$$

$$\Pr[G(k)=g]=\pi_{g}\text{ for }g=0,1$$

The group membership vector *G* is regarded as missing data for purposes of the “expectation/conditional maximization” algorithm (ECM). With a normal approximation, the complete data likelihood per observation *k* is:

$$\begin{matrix} \Pr[Y_{k},G(k)|\psi,V,\pi]=\Pr[Y_{k}|G(k)]\cdot\Pr[G(k)] \\ \propto\exp(-(Y_{k}-\psi_{G(k)})^{2}/2\tau_{G(k)k})\cdot(\tau_{G(k)k})^{-1/2}\cdot\pi_{G(k)} \end{matrix}$$

Now suppose that the variances are known (approximately). Define the free variable , and $\pi_{0}=1-\pi_{1}$, and similarly for the current fixed estimate . Following standard calculations, the expectation of the complete-data log likelihood is

$$\frac{\pi^{*}_{0k}}{\pi^{*}_{1k}}=\frac{\Pr(Y_{k},G(k)=0|\psi_{0}^{*},V_{0}^{*},\pi_{0}^{*})}{\Pr(Y_{k},G(k)=1|\psi_{1}^{*},V_{1}^{*},\pi_{1}^{*})}=\frac{\pi^{*}_{0}}{\pi^{*}_{1}}\frac{\exp\left(-(Y_{k}-\psi_{0}^{*})^{2}/2(V_{0}^{*}+\sigma_{k}^{2})\right)/\sqrt{V_{0}^{*}+\sigma_{k}^{2}}}{\exp\left(-(Y_{k}-\psi_{1}^{*})^{2}/2(V_{1}^{*}+\sigma_{k}^{2})\right)/\sqrt{V_{1}^{*}+\sigma_{k}^{2}}}$$

so that $E^{*}N_{g}=\sum_{k} \pi_{gk}^{*}$ for *g*=0,1.

To maximize *Q*, we set its partial derivatives to zero.

$$\partial Q/\partial\psi_{g}=-\sum_{k} \pi^{*}_{gk}\left((Y_{k}-\psi_{g})\left(\sigma^{2}_{k}+\hat{V}_{g}\right)^{-1}\right)=0$$

and

$$\partial Q/\partial V_{g}=\frac{1}{2}\sum_{k} \pi^{*}_{gk}\left(+(Y_{k}-\psi_{g})^{2}\left(\sigma^{2}_{k}+V_{g}\right)^{-2}-\left(\sigma^{2}_{k}+V_{g}\right)^{-1}\right)=0$$

We cannot solve these two equations simultaneously. However, fixing $V_{g}=\hat{V}_{g}$we can solve the first :

$$\hat{\psi}_{g}=\frac{\sum_{k} \pi^{*}_{gk}Y_{k}\left(\sigma^{2}_{k}+\hat{V}_{g}\right)^{-1}}{\sum_{k} \pi^{*}_{gk}\left(\sigma^{2}_{k}+\hat{V}_{g}\right)^{-1}}$$

and fixing $\psi_{g}=\hat{\psi}_{g}$we can solve the second:

$$\begin{matrix} \sum_{k} \pi^{*}_{gk}\left((Y_{k}-\psi_{g})^{2}-\left(\sigma^{2}_{k}+V_{g}\right)\right)=0 \\ \sum_{k} \pi^{*}_{gk}(Y_{k}-\psi_{g})^{2}=\sum_{k} \pi^{*}_{gk}\left(\sigma^{2}_{k}+V_{g}\right)=\sum_{k} \pi^{*}_{gk}\sigma^{2}_{k}+N_{g}^{*}V_{g} \\ \hat{V}_{g}=\max\left(0,\sum_{k} \pi^{*}_{gk}(Y_{k}-\hat{\psi}_{g})^{2}-\sum_{k} \pi^{*}_{gk}\sigma^{2}_{k}\right)/N_{g}^{*} \end{matrix}$$

So in the M step we iterate between these formulas for $\hat{\psi}_{g}$ and for $\hat{V}_{g}$. This constitutes an ECM algorithm.

The ECM context makes it easy to account for constraints. In a mixture of correlation distributions, it may be assumed that . We might also want to assume , or at least to test it to see if the variation in the *g*=0 component is due only to noise. These constraints are inserted into the respective formulas.
